# Mr. Warner, on Hooping Cough

**Published:** 1802-04

**Authors:** W. Warner

**Affiliations:** Member of the Royal College of Surgeons. Victualling Office Square, Tower Hill


### *To the Editors of the Medical and Phyſical Journal.*

GENTLEMEN,

IF the encloſed be deemed worthy of inſertion in your very valuable Journal, I beg leave to offer it for the ſame.

Your's, &c.

*Victual'ing Office Square, Tower Hill.*

W. WARNER,
Member of the Royal College of Surgeons.

---

MUCH has been ſaid in your eſteemed Journal on the beneficial effects derived from the external application of Opium in ſeveral complaints of the human body; and its uſe has been ſtrikingly beneficial in relieving patients from the excruciating pain of exceſſive cramp in the bowels and lower extremities, attendant on an alarming diſeaſe, (when neglected by patients, and ſuffered to continue without calling in medical aſſiſtance) viz. Cholera Morbus; and when Opium has been ejected immediately on being taken into the ſtomach, as I have ſeveral times witneſſed.

Of all the periods of life, none ſeem to demand our particular attention to relieve the many afflictions thereof ſo much as that of Childhood. Enough cannot be ſaid in praiſe of the diſcovery made of the Cow-pox, when we conſider the dreadful malady, with all its fatal ſymptoms, which moſt frequently attack the above period, and which will be now thoroughly overcome.

Another diſeaſe moſt incident to the aforementioned period, and very diſtreſſing to the ſufferer as well as tedious in its duration, by which very many are deſtroyed, is the diſeaſe termed Hooping Cough.

Having been the ſubject of this diſeaſe at an adult age, my thoughts were often engaged with a ſtrong deſire to relieve (I will not ſay cure) in future caſes ſpeedily. Nothing ſeemed ſo likely as the uſe of Opium; but it being dangerous to be put into the hands of patients, and opportunity not offering for its uſe, it being a common received opinion that no remedy will ſucceed but change of air, I almoſt relinquiſhed the idea, till hearing that four patients, whom I knew had it ſeverely, were relieved by the uſe of opium alone. (It was recommended by a perſon not of the profeſſion.)

An opportunity now offering in my own family, an adult ſubject, at my requeſt he made uſe of the remedy; at the ſame

 time time it was applied to a patient between two and three years of age. Both ſubjects being very different in all circumſtances, I will take the liberty of relating both caſes.

S. B. 21 years of age, a healthy conſtitution and ſanguine temperament, was attacked with cough, ſuppoſed from taking cold, which increaſed in violence about three weeks, during which time a variety of balſamic medicines, with anodynes, were taken, but to no good effect. Soon, from its violence, it became alarming; the fits of coughing were attended with hæmorrhage from the noſe, and terminated either with vomiting, or a ſtate almoſt amounting to ſuffocation; which left no doubt of its being hooping cough: A violent ſoreneſs of the abdominal muſcles was occaſioned by its urgency; and debility, to a great degree, was experienced.

Fourteen ounces of blood were drawn from the arm, which ſhewed no ſigns of inflammatory affection, but in every reſpect in a healthy ſtate. Liquid laudanum was rubbed all over the abdomen and pit of the ſtomach, twice a day, (without taking any medicine) which gave him great relief; the cough abated in frequency and violence; vomiting and hæmorrhage both ceaſed. This was continued for about a fortnight, when the cough almoſt totally ſubſided, and his uſual ſtate of health returned.

The next caſe was a ſubject very unpromiſing for ſo violent a diſeaſe, and in whom opium was alſo ſerviceable.

I. P. between two and three years of age, a weakly habit, much inclined to rickets and ſcrofula, was attacked with hooping cough; and when I firſt ſaw him, very unfavourable ſymptoms were preſent; the cough almoſt inceſſant, breathing very difficult, countenance ſwollen and rather livid, very feveriſh, and refuſal of all food; no ſleep, but comatoſe. The laudanum was uſed as in the former caſe; and, in addition, ſmall doſes of the decoction of the yellow bark were given three times a day, and the bowels now and then relieved by a gentle purgative. Good effects were viſible; in a few days the above ſymptoms went off, and the appetite returned. The bark was left off, and the laudanum uſed for about a fortnight longer, when the child was recovered.

Another, in the ſame family, only eight months old, is now under the uſe of the ſame remedy, with only a laxative now and then, and is recovering.*

Dr. Cullen in his excellent work, (vol. 3,) ſpeaking of this diſeaſe ſays, "When the contagion is recent and continues to act

* Nine or ten caſes have ſucceeded with uſing this remedy.

act, we neither know how to correct or how to expel it; and therefore the difeafe neceffarily continues for fome time: but it is probable that the contagion in this, as in other inftances, ceafes at length to act; and that then the difeafe continues, as in other convulfive affections, by the power of habit alone."

After directing various means, according to different ftages of the complaint, he mentions the feveral antifpafmodics; he relies moft on opium, but there I prefume is meant the internal ufe; whereas, we know the uncertainty of the dofe, as well as the inconvenient effects produced by it in children as well as in adults. On the other hand, its external ufe promifes the greateft benefit.

Many remedies are ufed for this difeafe, but the one moft ufed, is an outward application, called Roche's Embrocation, which, in three inftances, ill effects appeared to arife from the cough fuddenly ftopping after the ufe of it; two died in ftrong convulfions, the third was relieved from approaching fuffocation by a fmart purge and an emetic, and is now living.

The laudanum moderates each paroxyfm in frequency, &c. therefore, if early ufed, prevents the expectoration which is otherwife caufed, and though the cough goes on fome time, it evidently fhortens the ufual paroxyfm. In order to difguife the laudanum, a few drops of æther may be added, and in this way ufed as an embrocation twice or thrice a day, over the ftomach and belly.

A flannel waiftcoat fhould be worn by the patient, as, no doubt, it promotes abforption, and prevents the viciffitudes of the climate taking that effect on the fkin, which we know it does, and acts as an exciting caufe of coughing.

Perhaps I am premature in offering this to the public notice; but the difeafe being fo very prevalent at prefent, and confequences fo generally fatal, I thought it might excite others in the profeffion to make trial of the fame remedy; and if with the fame fuccefs, I hope it will plead a fufficient apology.

